# Eating Disorders and Autistic Traits Camouflaging: Insights from the EAT Study

**DOI:** 10.3390/nu18010034

**Published:** 2025-12-21

**Authors:** Maddalena Cesco, Marco Garzitto, Veronica Croccia, Francesca Bier, Luana Saetti, Matteo Balestrieri, Marco Colizzi

**Affiliations:** 1 Unit of Psychiatry and Eating Disorders, Department of Medicine (DMED), University of Udine, 33100 Udine, Italy; cesco.maddalena@spes.uniud.it (M.C.); marco.garzitto@uniud.it (M.G.); croccia.veronica@spes.uniud.it (V.C.); bier.francesca@spes.uniud.it (F.B.); luana.saetti@uniud.it (L.S.); matteo.balestrieri@uniud.it (M.B.); 2 Department of Psychosis Studies, Institute of Psychiatry, Psychology and Neuroscience, King’s College London, London SE5 8AF, UK

**Keywords:** eating disorders, clinical characterization, mental health, prevention, autism-spectrum disorder

## Abstract

Background: Feeding and eating disorders (FEDs) often present in comorbidity with other psychiatric conditions, with a growing body of evidence underscoring their association with autism spectrum disorder (ASD). Individuals with ASD or significant autistic traits (ATs), especially females, often engage in camouflaging strategies to mask their symptoms. However, empirical research on the role of camouflaging within this association is still emerging. This study aimed to assess the prevalence of ATs in individuals with FEDs and to examine their connection with psychological well-being, along with the role of camouflaging as a potential mediator in this association. Methods: A total of 131 individuals with FEDs were assessed through a medical record review, a socio-demographic form, and self-administered questionnaires evaluating FEDs symptoms (EDI-3) and ASD-related features (RAADS-R, AQ, EQ, CAT-Q). Results: In total, 16% of patients scored above the possible high ATs in clinical settings (whereas 53% exceeded the original cut-off) and 25% showed significant camouflaging, without differences between FED diagnoses. ATs were associated with both FED symptom severity and general maladjustment. Importantly, the latter was not directly explained by ATs themselves, but was mediated separately by camouflaging and FED symptomatology. After statistical adjustments, the parallel mediating pathways contributed similarly (48% and 52%). Conclusions: A considerable subset of individuals with FEDs presents significant ATs, with camouflaging arguably linked to psychological distress through a pathway parallel to that of FED symptomatology. This overlap between FEDs and ASD may be clinically meaningful, highlighting the potential importance of assessing ATs and camouflaging to support personalized diagnostic and therapeutic interventions.

## 1. Introduction

Feeding and eating disorders (FEDs) constitute a group of severe psychiatric conditions marked by persistent disturbances in eating behaviours or weight regulation strategies. Central to their development and persistence are maladaptive cognitions and attitudes regarding body weight, shape, and food. They exert a profound impact on physical health and significantly compromise psychosocial functioning across various domains of life [1,2]. Individuals diagnosed with bulimia nervosa (BN) or binge-eating disorder (BED), demonstrate substantially greater impairments in emotional well-being. On the other hand, individuals diagnosed with anorexia nervosa (AN) exhibit higher rate of depression, self-harming behaviours, and suicidal ideation [3]. Moreover, social difficulties have been identified as both risk factors and maintaining mechanisms in the development and persistence of FEDs [4].

FEDs are estimated to affect between 2% and 5% of individuals over their lifetime and worldwide, with a markedly higher prevalence observed among females compared to males [5]. Psychiatric comorbidities are highly prevalent among individuals with FEDs, affecting over 70% of cases. The most frequently co-occurring conditions in adult population include mood and anxiety disorders, obsessive–compulsive disorders, substance use disorders, and personality disorders [6,7,8]. A growing body of evidence has also underscored the association between FEDs and key neurodevelopmental conditions, such as autism spectrum disorder (ASD) [9,10,11].

ASD is defined by enduring impairments in social communication and interaction across diverse settings, along with the presence of restricted and repetitive behaviour, interests, or activities. The presentation of the disorder is highly heterogeneous and influenced by multiple factors, including the severity of autistic features, developmental stage, age, and possibly gender, thus justifying the conceptualization of ASD as a spectrum condition [1]. Furthermore, subthreshold autistic traits (ATs) are recognized in the general population and represent manifestations that are qualitatively similar, though milder in severity, to the clinical features of ASD. They are suggested to underlie various subclinical symptoms, isolated behavioural features, personality characteristics, and temperamental traits, thereby constituting a vulnerability factor for the development of psychiatric disorders [12].

ASD is identified in males three to four times more frequently than in females, with females typically receiving a diagnosis at a later age. However, females with ASD often present with more subtle symptoms, such as stronger social communication skills, less conspicuous repetitive behaviours, and socially typical special interests [1]. They also often engage in camouflaging strategies to mask their ASD symptoms, which can make their difficulties less visible and contribute to the risk of underdiagnosis or misdiagnosis [1,13,14,15]. Camouflaging behaviours have been associated with specific patterns of FEDs, such as orthorexia and avoidant/restrictive food intake disorder (ARFID) [16,17]. In adolescent individuals diagnosed with AN, an association has been observed between interoceptive deficits, ATs, alexithymia, and camouflaging behaviours, highlighting the difficulties individuals in the autism spectrum experience in processing internal bodily signals [18]. Moreover, camouflaging has been addressed as a significant predictor of FED symptomatology in adult individuals with ASD [19] and has been linked to the presence and severity of FED symptoms [20]. However, empirical evidence supporting the relationship among FEDs, ASD, and camouflaging remains limited. In particular, the influence of this interplay, especially the role of camouflaging, on overall psychological well-being has yet to be thoroughly explored, underscoring the need for further research.

Based on these premises, the present study sought to extend existing evidence on the prevalence of significant ATs in individuals with FED symptomatology and the use of camouflaging strategies. The primary aim was to investigate the relationship between ATs and psychological well-being, while assessing the mediating roles of FEDs’ severity and camouflaging behaviours through a proposed mediation model.

## 2. Materials and Methods

### 2.1. Study Design and Participants

An observational study was conducted at the University Hospital of Udine, involving patients receiving care at the One-stop centre for the treatment of FEDs at the Unit of Psychiatry and Eating Disorders (Italian: CUDICA, Centro Unico per il trattamento dei DIsturbi del Comportamento Alimentare). The inclusion criteria comprised proficiency in Italian, a DSM-5 diagnosis of FED, and provision of informed consent. Patients who did not complete the proposed main self-assessments were excluded from the study. This study was conducted in accordance with the Declaration of Helsinki and approved by the Institutional Review Board of the Department of Medicine (DMED) at the University of Udine (133/2023).

### 2.2. Assessment

Clinical and socio-demographic data were obtained through a review of medical records and the completion of a standardized form. All participants underwent psychometric assessment to evaluate symptoms related to FED and features associated with ASD.

#### 2.2.1. Eating Disorder Inventory, Version 3 (EDI-3)

The EDI-3 is a validated self-report measure assessing FED symptoms and related psychological features, widely used for early risk identification and treatment monitoring [21,22]. In this study, the Italian validated version was used [23], applying Italian norms. The questionnaire includes 91 items across 12 primary scales: three are FEDs-specific, nine are psychological scales, and three are validity scales. The psychological scales are further grouped into four composite scales (Ineffectiveness, Interpersonal Problems, Affective Problems Composite, and Overcontrol). The instrument also yields two global indices: the Eating Disorder Risk Composite (EDRC), reflecting FEDs symptom severity, and the General Psychological Maladjustment Composite (GPMC), summarizing overall psychological distress.

#### 2.2.2. Ritvo Autism and Asperger Diagnostic Scale, Revised Version (RAADS-R)

The RAADS-R is an 80-item self-report instrument developed to support the diagnosis of ASD in adults with average or above-average intellectual functioning. It has demonstrated high specificity (100%) and sensitivity (97%), along with strong internal consistency and test–retest reliability. The RAADS-R comprises four core symptom domains: social relatedness, circumscribed interests, language, and sensory–motor. A total score of 65 or above is considered indicative of a possible ASD [24], although this cut-off has been considered too prone to false positives in clinical settings. A cut-off score of 120 has been suggested as more appropriate for application in mental health services [25], providing a balance of acceptable sensitivity (75%) and specificity (71%). The Italian version of the questionnaire, developed by Moscone and Vagni in 2013 [26], was employed in the present study as main measure of significant ATs.

#### 2.2.3. Autism Spectrum Quotient (AQ)

The AQ is a 50-item self-report screening instrument, validated in its Italian version, designed to measure the extent to which individuals with average intellectual functioning exhibit traits associated with ASD [27,28]. The items cover five domains: social skills, attention switching, attention to detail, communication, and imagination. A total score of 32 or above is considered indicative of clinically significant ATs; however, it does not constitute a diagnosis. In this study, the Italian version of AQ was administered as a supplementary measure of ATs.

#### 2.2.4. Empathy Quotient (EQ)

The EQ is a 40-item self-administered questionnaire designed to assess empathy in adults with average or above-average intellectual functioning [29,30]. A score of 30 or below is considered a useful cut-off for distinguishing individuals with low empathy. Adults with ASD and average or high intellectual functioning typically score significantly lower on the EQ compared to neurotypical controls. In this study, the Italian version of EQ was administered as a supplementary measure of ATs.

#### 2.2.5. Camouflaging Autistic Traits Questionnaire (CAT-Q)

The CAT-Q is a valid and reliable self-report instrument for assessing camouflaging behaviours in adults [31]. The behaviours associated with camouflaging can be categorized into three strategies: masking (efforts to present a non-autistic or less visibly autistic persona), compensation (techniques used to manage or overcome social and communication difficulties), and assimilation (behaviours aimed at adapting to or blending into uncomfortable social situations [31,32]. The CAT-Q includes 25 items, and a total score above or equal to 100 is considered the cut-off for a significant use of camouflaging. In its Italian version, adopted in this study, it has proven to be a valuable instrument for assessing ASD camouflaging [33]. We preferentially used standards from Italian validation.

### 2.3. Data Analysis

Initial univariate analyses were conducted with a between-observations *t*-test with Welch correction, a χ^2^-test with bootstrapping re-sampling (25,000 replication), and a test of Pearsons correlation coefficient. Where needed, correction for potential confounders were performed with multiple linear or multinominal regression models. Models were tested with the F-test or with the Wald-χ^2^-test, respectively. Mediations were conducted in PROCESS-5.0 framework [34], specifying a series of parallel mediation analyses (Model-4). Measures were entered in their original units, without centring. All mediation analyses included a correction for measurement error [35], with the following reliabilities calculated in the sample using Cronbach coefficient: 0.939 (RAADS-R), 0.924 (CAT-Q), and 0.840 (EDRC). A reliability of 0.950 was assumed for BMI and of 0.999 for age at assessment. Confidence intervals for indirect effects were calculated with the bootstrapping procedure (with 25,000 samples). Missing data were handled using pairwise deletion in univariate and preliminary analyses. There were no missing data in the mediation analyses. Statistical significance was set at α = 0.050 (two-tailed), also for confidence intervals (i.e., 95%). All analyses were conducted in R-4.5.1 [36]. PROCESS-5.0 macros for R were used for mediations [37].

## 3. Results

### 3.1. Sample Description and Clinical Features

One hundred and thirty-one patients with a diagnosis of FED were recruited, who were predominantly females (89.3%), with ages ranging from 18 to 64 years (mean: 33.2 ± 14.43 years old). They all were Italian, except for one. The FED diagnoses, in order of frequency, were as follows: binge-eating disorder (BED; 29.0% of the sample); anorexia nervosa (AN; 25.2%); other specified feeding or eating disorder (OSFED; 22.1%); bulimia nervosa (BN; 18.3%); and unspecified feeding or eating disorder (UFED; 5.3%). The AN diagnostic category included both individuals with a restricting sub-type (AN-R; 15.3% of the total sample; 60.6% of the patients with AN) and with a binge-eating/purging sub-type (AN-BP; 9.9%). Socio-demographic and clinical characteristics of the sample are reported in Table 1, with statistically significant differences between FED diagnoses.

The diagnostic groups statistically significantly differed in age at assessment (F_5,125_ = 8.06, *p* < 0.001). Overall, after adjusting for sex and age, differences were significant for BMI (F_5,123_ = 14.32, *p* < 0.001) and previous hospitalisations for FEDs (LR-χ^2^5 = 16.20, *p* = 0.006). EDI-3 global indices were also different between groups: EDRC (F_5,123_ = 10.62, *p* < 0.001) and GPMC (F_5,125_ = 4.13, *p* = 0.002). Details are reported in the Supplementary Materials (Table S1).

### 3.2. Effects of ATs and Camouflaging

The 16.0% of patients scored above the RAADS-R suggested the threshold for possible ASD in mental health service populations (53.4% when considering the original RAADS-R cut-off; 10.2% based on the AQ cut-off; and 12.5% based on the EQ cut-off) and the 25.2% of them above the CAT-Q cut-off suggested for significant camouflaging. The level of ATs (i.e., RAADS-R score; see Table 1) was positively associated at statistically significant level with having had school failures (F_1,65_ = 4.18, *p* = 0.045) and with having psychiatric (F_1,127_ = 10.61, *p* = 0.001) and severe non-psychiatric (F_2,101_ = 4.01, *p* = 0.021) comorbidities. Also, ATs were higher in participants who were prescribed or had been prescribed antidepressants (previous: F_1,125_ = 16.37, *p* < 0.001; current: F_1,123_ = 11.92, *p* = 0.001) or antipsychotics (F_1,125_ = 5.64, *p* = 0.019; F_1,123_ = 9.72, *p* = 0.002).

As expected, ATs and camouflaging (i.e., CAT-Q standard z-score) were correlated (r = +0.474, with a 95% confidence interval in [+0.330, +0.597] range and *p* < 0.001). ATs predicted the 22.5% of the use of camouflaging (adjusted-R^2^ = 0.219; B = +0.327, *p* < 0.001) and the effect was similar after correcting for age at assessment and BMI (R^2^ = 0.262, adjusted to 0.244; B = +0.330, *p* < 0.001; for age: −0.345, *p* = 0.037; and for BMI: −0.046, *p* = 0.859). Also, the CAT-Q standard z-score was negatively correlated with age at assessment (r = −0.172, *p* = 0.049).

As reported in Table 1, none of the comparisons between diagnostic groups for ASD-related measures reached statistical significance, neither when using continuous scores nor considering cut-offs. Instead, ASD-related measures were significantly associated with all standardized EDI-3 psychological scales, in a positive direction (Table 2). Considering the overall summary scales, similar correlations were observed for the GPMC scale (respectively, +0.373 vs. +0.426, all with *p* < 0.001), while only the RAADS-R score significantly correlated with the EDRC scale (r = +0.205, *p* = 0.019).

### 3.3. Mediation Analysis

**Figure 1:**
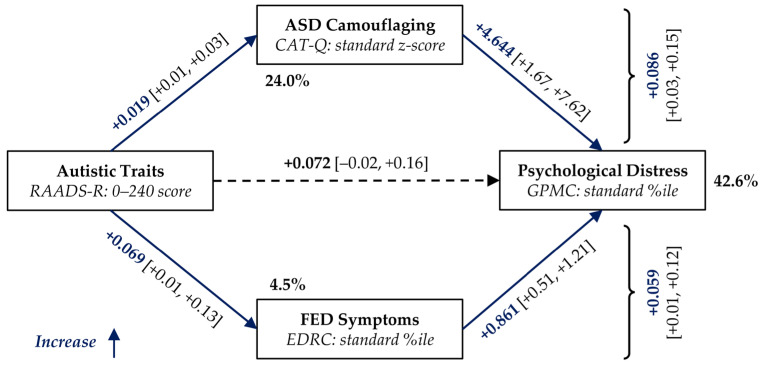
Parallel mediation diagram of the effect of RAADS-R score on GPMC scale (EDI-3). CAT-Q score and EDRC scale (EDI-3) were included as mediators. %ile: Percentile; ASD: autism spectrum disorder; CAT-Q: Camouflaging Autistic Traits Questionnaire; EDI-3: Eating Disorder Inventory, version 3; EDRC: Eating Disorder Risk Composite scale (EDI-3); FED: feeding or eating disorder; GPMC: General Psychological Maladjustment Composite scale (EDI-3); and RAADS-R: Ritvo Autism Asperger Diagnostic Scale, Revised version. Bold percentages indicate the variance mediated by the corresponding regression. Bold coefficients and solid lines in red/blue indicate statistically significant effects (*p* < 0.050). The 95% confidence intervals for coefficients are reported in square brackets. Curly braces denote the indirect effects via the corresponding mediator.

A first parallel mediation analysis was undertaken to examine the indirect, simultaneous effect of the CAT-Q and EDRC scores on the direct positive association between the RAADS-R and GPMC scores. The complete model for the GPMC score was statistically significant (F_3,127_ = 19.47, *p* < 0.001). As reported in Figure 1, the effects of CAT-Q (*p* = 0.003) and EDRC (*p* < 0.001) on GPMC were significant, but that of RAADS-R was not (*p* = 0.126), suggesting an indirect effect (B = +0.146, with 95% ci: [+0.065, +0.237]) that accounted for the 66.9% of the total effect. The effects of both mediators were confirmed, with CAT-Q accounting for the 59.1% of the indirect effect and EDRC for the 40.9%.

**Figure 2:**
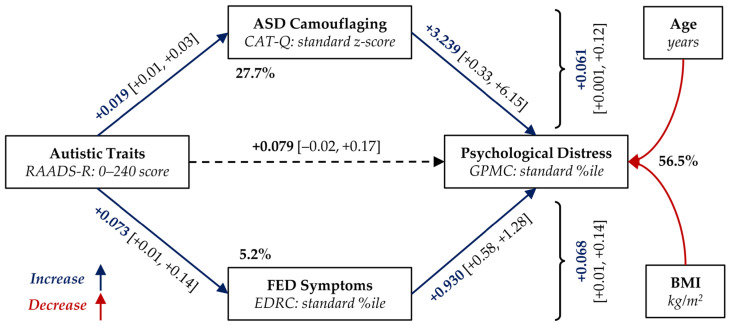
Parallel mediation diagram of the effect of RAADS-R score on GPMC scale (EDI-3). CAT-Q and EDRC scale (EDI-3) were included as mediators. All analyses were adjusted for potential confounders: age at assessment and BMI. %ile: Percentile; ASD: autism spectrum disorder; BMI: body mass index; CAT-Q: Camouflaging Autistic Traits Questionnaire; EDI-3: Eating Disorder Inventory, version 3; EDRC: Eating Disorder Risk Composite scale (EDI-3); FED: feeding or eating disorder; GPMC: General Psychological Maladjustment Composite scale (EDI-3); and RAADS-R: Ritvo Autism Asperger Diagnostic Scale, Revised version. Bold percentages indicate the variance mediated by the corresponding regression. Bold coefficients and solid lines in red/blue indicate statistically significant effects (*p* < 0.050). The 95% confidence intervals for coefficients are reported in square brackets. Curly braces denote the indirect effects via the corresponding mediator.

In a second mediation, the participants’ age at assessment and BMI were introduced in the linear models as potential confounders. The introduction of these covariates did not substantially change the results (Figure 2; complete model: F_5,125_ = 20.64, *p* < 0.001), resulting in a modest decrease in total indirect effect (+0.129 [+0.042, +0.220]), but with different effects on single paths. In fact, the indirect path through the CAT-Q was substantially reduced (47.2% of the indirect effect) and that through the EDRC increased (52.7%). Both age and BMI had a statistically significant negative effect on the GPMC score (respectively, −0.289 [−0.527, −0.046], *p* = 0.020; −0.609 [−0.998, −0.220], *p* = 0.002). Age also showed a near-significant trend toward reducing the CAT-Q score (*p* = 0.053).

Finally, a series of parallel mediation analyses were conducted on diagnostic groups, both without covariates and with correction for age and BMI (see Figure S1 in the Supplementary Materials). For feasibility reasons, AN-R and AN-BP diagnoses were merged into a single group (AN), as were OSFED and UFED (Other). Given the small sample sizes involved, these analyses should be regarded as purely exploratory and are intended solely to qualitatively evaluate potential heterogeneity across diagnoses. The observed effects substantially overlapped with those of the overall sample for the BED group and confirmed the direction of the effects for AN and Other groups (albeit without statistical significances). Unexpectedly, BN patients showed an inverted direction for the indirect paths.

## 4. Discussion

The present study aimed to estimate the prevalence of ATs in a sample of patients with FEDs and to examine the associations among FEDs symptomatology, ATs, and camouflaging behaviours, with a focus on how this interplay affects psychological well-being.

Our sample comprised 131 patients; 16.0% exceeded the RAADS-R threshold for possible ASD in clinical settings, and 53.4% when considering the original cut-off. These findings were consistent with the results from the AQ and EQ, which identified 10.2% and 12.5% of participants, respectively, as presenting significant ATs. Previous evidence indicated a lifetime prevalence of ASD among individuals with FEDs of approximately 4.7% [10] and a prevalence of ASD-related symptoms among both adolescent and adult patients with AN ranging from 4% to 52.5% [38]. The RAADS-R score was then positively associated with the GMPC scale of the EDI-3, which is indicative of general psychological maladjustment [22], suggesting that the presence of ATs may be linked to the reduced overall psychological well-being of FED patients. Indeed, ATs have been shown to be stable over time, unrelated to body weight [9], associated with less favourable treatment outcomes, and resulting in more intensive care [38]. ATs were also associated with an increase in FEDs symptomatology (i.e., EDRC scale of the EDI-3). These associations are supported by biological and genetic investigations, suggesting that the ASD Polygenic Risk Score (PGS) increases the risk of developing AN. Moreover, a higher risk of developing AN has also been found among individuals with a full or maternal half sibling diagnosed with ASD [39]. In our sample, we did not observe any significant associations between specific FEDs’ diagnoses and RAADS-R scores, suggesting possible transdiagnostic effects. It remains challenging to clarify the complexity of these associations, which can also be examined from the RDoC perspective, according to which ATs would correlate with specific biobehavioural domains rather than categorical diagnoses [40]. Notably, preclinical models of ASD highlight an altered relationship between central homeostatic and hedonic neural circuits involved in food intake regulation [41], offering initial hints about the neurobiological underpinnings of eating-related symptoms in ASD.

To better explain these associations, we investigated the role of camouflaging behaviours in our study population. Overall, ASD camouflaging was reported by the 25.2% of the participants. Camouflaging showed a significant positive correlation with ATs, confirming its pertinence as a socio-behavioural response to ASD-related symptoms, predominantly being a characteristic of ASD females [1,5]. Notably, it is generally aimed at enhancing social adaptation, despite often resulting in increased subjective distress rather than improved well-being [42]. Thus, the observation of a relatively high frequency of camouflaging among our patients naturally prompts consideration of its potential adverse effect in terms of clinical presentation.

Following these lines, we proposed that both camouflaging and FED severity could mediate the relationship between ATs and patients’ overall well-being (GMPC scale of the EDI-3). Our main findings supported such hypotheses, with indirect paths through the CAT-Q score and the EDRC scale that were found to be largely independent. Without correction, the effect seems to be mediated mainly by camouflaging (59.1% vs. 40.9%), which was also more associated with ATs (variance explained: 24.0% vs. 4.5%, respectively). However, accounting for age differences between patients, the two paths were more balanced (47.2% vs. 52.7%). Some preliminary analyses indicated that the mechanism observed may be common to various FED diagnoses, but with some important inhomogeneities (e.g., BN group). However, the small size of the individual groups must be considered as limiting generalizability.

Although the available evidence remains limited, previous studies have reported an association between camouflaging and FED symptoms [19,20]. Our mediation results are consistent with and extend these findings. Specifically, we suggest that patients with elevated ATs experience an overall psychopathological burden linked to the parallel and separate contributions of FED symptoms and camouflaging behaviours. This pattern may indicate a distinctive psychopathological mechanism characterizing a subset of individuals with FEDs. Such a mechanism appears to be associated with a substantial impact on quality of life, as reflected in our sample, which showed marked subjective distress and notable repercussions across specific domains of health and functioning (e.g., school failures, comorbidities, and antidepressant or antipsychotic prescription). Considering these findings, together with the tendency toward chronicity and relapse in FEDs, especially in AN [5], our results offer a novel lens through which to understand the most complex clinical cases. This perspective underscores the need for more in-depth investigations, including longitudinal approaches. Accordingly, it may also help inform clinical strategies that extend beyond eating disorder-specific symptomatology and avoid attributing outcomes solely to ATs.

The present study has several strengths, including its moderate-sized sample and the use of internationally validated and standardized questionnaires. Furthermore, the data were collected from a third-level medical facility specialized in treatment of FEDs, allowing for an accurate clinical characterization of the sample. The service admits patients from a wide catchment area, favouring the selection of a sample that is representative of the general clinical population.

However, several limitations should be acknowledged. First, the cross-sectional design prevents longitudinal observation and limits causal inference as well as the formulation of subsequent clinical recommendations. Second, recruitment from a single psychiatric service and the predominantly female sample restricts the generalizability of the findings. Additionally, most assessments relied on self-report measures, and a full diagnostic evaluation for ASD was not conducted. Finally, potential comorbidities and the effects of psychopharmacological treatments were not systematically assessed.

## 5. Conclusions

Given the present findings, future research should aim to further investigate the comorbid aspects between ASD and FEDs, employing larger samples and longitudinal designs. Coping with the effects of ASD symptoms and/or a high level of ATs may be an important mechanism in the development or maintenance of psychological distress. Additionally, based on preliminary observations, a more in-depth comparison between different FEDs diagnoses could be of primary interest, to elucidate potentially differentiated psychopathological processes (even in the absence of simple differences in symptom expression). Such efforts may contribute to a more comprehensive understanding of the shared mechanisms underlying these conditions and support the development of more targeted therapeutic strategies.

## Figures and Tables

**Table 1:** Socio-demographic and clinical characteristics of the sample. Effects of FED diagnosis and RAADS-R total score are reported.

Patients (*N* = 131)	*N (%)* or *Mean ± SD [Min, Max]*		Diagnosis	RAARDS-R
FED diagnosis	*AN-R:*	20 (15.3%)	-	
	*AN-BP:*	13 (9.9%)		
	*BN:*	24 (18.3%)		
	*BED:*	38 (29.0%)		
	*OSFED:*	29 (22.1%)		
	*UFED:*	7 (5.3%)		
Sex	*Females:*	117 (89.3%)		
Age at assessment	*[years]*	32.8 ± 14.51 [17, 64]	*	
Schooling ^(6)^	*Middle school:*	30 (24.0%)		
	*High school:*	63 (50.4%)		
	*Degree or more:*	32 (25.6%)		
School failures ^(62)^	*Any:*	24 (34.8%)		↑
Relationship	*Single:*	88 (67.2%)		
	*Couple:*	43 (32.8%)		
Housing ^(1)^	*Old-family:*	67 (51.5%)	(*)	
	*New-family:*	34 (26.2%)		
	*Alone:*	24 (18.5%)		
	*Other:*	5 (3.9%)		
Current occupation ^(1)^	*Employed:*	75 (57.8%)	(*)	
	*Student:*	41 (31.5%)		
	*House-working:*	3 (2.3%)		
	*Unemployed:*	8 (6.2%)		
	*Retired:*	3 (2.3%)		
Age at FED onset ^(8)^	*[years]*	17.9 ± 9.15 [5, 53]		
Duration of FED ^(8)^	*[years]*	14.2 ± 14.08 [0, 57]	(*)	
BMI	*[kg/m^2^]*	25.9 ± 9.13 [13.2, 53.7]	*	
Previous hospitalizations ^(1)^	*Any (for FED):*	13 (10.0%)	*	
Psychiatric comorbidity	*Any (non-FED):*	48 (36.6%)		↑
Previous support ^(1)^	*Any (mental health):*	92 (70.8%)		
Non-psychiatric comorbidity	*Any:*	66 (50.4%)		
	*Chronic:*	62 (50.4%)		
	*Severe:*	12 (11.3%)	(*)	↑
Antidepressant ^(2,4)^	*Previous:*	30 (23.3%)		↑
	*Current:*	45 (35.4%)		
Stabilizer ^(2,4)^	*Previous:*	8 (6.2%)		
	*Current:*	11 (8.7%)		
Antipsychotics ^(2,4)^	*Previous:*	10 (7.8%)		↑
	*Current:*	17 (13.4%)		
Benzodiazepines ^(2,4)^	*Previous:*	19 (14.7%)		
	*Current:*	24 (18.9%)		
Other drugs ^(2,4)^	*Previous:*	23 (17.8%)		
	*Current:*	32 (25.2%)		
**EDI-3 scales**				
*Eating Disorder Risk Composite*	*[standard %ile]*	85.9 ± 12.43 [39, 99]	*	↑
*Global Psychological Maladjustment*	*[standard %ile]*	67.0 ± 21.56 [1, 99]	*	↑
**RAADS-R (with sub-scales)**				
Possible ASD (above cut-off)	*Original version (≥65):*	70 (53.4%)		-
	*Clinical setting (≥120):*	21 (16.0%)		-
*Total score*	*[score: 0, 240]*	74.0 ± 39.36 [7, 168]		-
*Social relatedness scale*	*[score: 0, 117]*	36.4 ± 20.92 [3, 98]		-
*Circumscribed interests scale*	*[score: 0, 42]*	16.3 ± 8.67 [0, 41]		-
*Language scale*	*[score: 0, 21]*	4.8 ± 4.19 [0, 15]		-
*Sensory–Motor scale*	*[score: 0, 60]*	16.5 ± 11.92 [0, 50]		-
**CAT-Q (with sub-scales)**				
Camouflage (above cut-off)	*High score (≥100):*	33 (25.19%)		↑
*Total score*	*[standard z]*	+0.1 ± 1.44 [−2.7, +3.2]		↑
*Compensation score*	*[standard z]*	−0.3 ± 1.19 [−1.8, +3.2]		↑
*Masking score*	*[standard z]*	<+0.1 ± 1.37 [−2.8, +2.9]		↑
*Assimilation score*	*[standard z]*	+0.4 ± 1.38 [−2.2, +3.6]		↑
**Other ASD-related**				
*Adult Autism Spectrum Quotient* ^(13)^	*[score: 0, 50]*	20.5 ± 7.78 [6, 42]		↑
*Empathy Quotient* ^(3)^	*[score: 0, 80]*	44.9 ± 11.89 [9, 70]		↑

%ile: Percentile; AN: anorexia nervosa; ASD: autism spectrum disorder; BED: binge-eating disorder; BMI: body mass index; BN: bulimia nervosa; -BP: binge-eating/purging sub-type of AN; CAT-Q: Camouflaging Autistic Traits Questionnaire; EDI-3: Eating Disorder Inventory, version 3; FED: feeding or eating disorder; Max: maximum observed value; min: minimum observed value; N: number of observations; OSFED: other specified FED; Other: diagnosis group including OSFED and UFED; -R: restrictive sub-type of AN; RAADS-R: Ritvo Autism Asperger Diagnostic Scale, Revised version; SD: standard deviation; and UFED: unspecified FED. *: Comparison between diagnosis groups showed a statistically significant difference (*p* < 0.050); (*): comparison between diagnosis group was no more statistically significant (*p* ≥ 0.050) after the correction for sex and age; ↑: the measure was positively associated with RAARDS-R total score at statistically significant level (*p* < 0.050), also after correction for sex and age; -: the test cannot be performed; and ^(N)^: the numbers of missing observations are indicated in superscript, in parentheses.

**Table 2:** Associations between score in RAADS-R and standard score in CAT-Q (in z-scores) with main confounders (i.e., sex, age at assessment, and BMI) and standard scores in EDI-3 scales (in percentiles).

	RAADS-R	CAT-Q
Sex	t_16.1_ = −0.65	t_17.4_ = 0.71
Age at assessment	r = +0.040 [−0.133, +0.210]	r = −0.172 [−0.334, −0.001] *
BMI	r = −0.127 [−0.292, +0.046]	r = −0.163 [−0.326, +0.009]
**EDI-3-specific scales**		
*Eating Disorder Risk Composite*	r = +0.205 [+0.035, +0.364] *	r = +0.153 [−0.019, +0.316]
*Drive for Thinness*	r = +0.123 [−0.050, +0.288]	r = +0.168 [−0.004, +0.330]
*Bulimia*	r = +0.144 [−0.028, +0.308]	r = +0.039 [−0.134, +0.209]
*Body Dissatisfaction*	r = +0.202 [+0.031, +0.361] *	r = +0.092 [−0.081, +0.259]
**EDI-3 psychological scales**		
*Global Psychological Maladjustment*	r = +0.373 [+0.215, +0.512] *	r = +0.426 [+0.274, +0.557] *
*Ineffectiveness Composite*	r = +0.355 [+0.195, +0.496] *	r = +0.371 [+0.213, +0.510] *
*Interpersonal Problems Composite*	r = +0.429 [+0.278, +0.560] *	r = +0.364 [+0.205, +0.504] *
*Affective Problems Composite*	r = +0.316 [+0.153, +0.463] *	r = +0.353 [+0.193, +0.495] *
*Overcontrol Composite*	r = +0.287 [+0.122, +0.437] *	r = +0.439 [+0.290, +0.568] *
*Low Self-Esteem*	r = +0.298 [+0.134, +0.447] *	r = +0.318 [+0.155, +0.464] *
*Personal Alienation*	r = +0.359 [+0.200, +0.500] *	r = +0.385 [+0.229, +0.522] *
*Interpersonal Insecurity*	r = +0.411 [+0.257, +0.544] *	r = +0.312 [+0.149, +0.459] *
*Interpersonal Alienation*	r = +0.318 [+0.155, +0.465] *	r = +0.323 [+0.161, +0.469] *
*Interoceptive Deficits*	r = +0.288 [+0.122, +0.438] *	r = +0.300 [+0.135, +0.448] *
*Emotional Dysregulation*	r = +0.289 [+0.124, +0.439] *	r = +0.359 [+0.199, +0.499] *
*Perfectionism*	r = +0.234 [+0.066, +0.390] *	r = +0.300 [+0.136, +0.449] *
*Ascetism*	r = +0.261 [+0.093, +0.414] *	r = +0.435 [+0.285, +0.565] *
*Maturity Fears*	r = +0.204 [+0.034, +0.363] *	r = +0.205 [+0.035, +0.364] *

BMI: body mass index; CAT-Q: Camouflaging Autistic Traits Questionnaire; EDI-3: Eating Disorder Inventory, version 3; RAADS-R: Ritvo Autism Asperger Diagnostic Scale, Revised version. *: statistically significant (*p* < 0.050). The 95% confidence intervals for the Pearson correlations are reported in square brackets.

## Data Availability

The datasets used and/or analyzed during the current study are available from the corresponding author on reasonable request.

## Supplementary Materials

The following supporting information can be downloaded at: https://www.mdpi.com/article/10.3390/nu18010034/s1. Figure S1: Parallel mediation effects in the overall sample (N = 131) and within each diagnostic group. Direct prediction was of RAADS-R score on GPMC scale (EDI-3, in standard percentiles). Indirect effect includes both mediation effects of CAT-Q score (in standard z-score) and EDRC scale (EDI-3, in standard percentiles). On the left, analyses were not adjusted for any confounder. On the right, all analyses were adjusted for potential confounders: age at assessment (in years) and BMI (in kg/m^2^). Table S1: Socio-demographic and clinical characteristics of the sample, by diagnosis groups.

## Abbreviations

The following abbreviations are used in this manuscript:

%ile	Percentile
AN	Anorexia Nervosa
ASD	Autism Spectrum Disorder
ATs	Autistic Traits
BED	Binge-Eating Disorder
BMI	Body Mass Index
BN	Bulimia Nervosa
-BP	Binge-Eating/Purging Sub-Type of AN
CAT-Q	Camouflaging Autistic Traits Questionnaire
CUDICA	Centro Unico per il trattamento dei DIsturbi del Comportamento Alimentare [Italian; One-stop centre for the treatment of FEDs of the Unit of Psychiatry and Eating Disorders]
DSM-5	Diagnostic and Statistical Manual of Mental Disorders, 5th edition
EDI-3	Eating Disorder Inventory, version 3
EDRC	Eating Disorder Risk Composite scale (EDI-3)
FEDs	Feeding and Eating Disorders
GPMC	General Psychological Maladjustment Composite scale (EDI-3)
Max	Maximum Observed Value
min	Minimum Observed Value
N	Number of Observations
OSFED	Other Specified FED
Other	Diagnosis Group including OSFED and UFED
-R	Restrictive Sub-Type of AN
RAADS-R	Ritvo Autism Asperger Diagnostic Scale, Revised version
RDoC	Research Domain Criteria
SD	Standard Deviation
UFED	Unspecified FED
