# Supplementary material for: Eating Disorders and Autistic Traits Camouflaging: Insights from the EAT Study
Source: Nutrients. 2025 Dec 21;18(1):34. doi: 10.3390/nu18010034 (PMC12787464; doi:10.3390/nu18010034)
Supplement: Supplementary file 1 [file nutrients-18-00034-s001.zip › nutrients-4031551-supplementary.pdf]

Supplementary Materials

Table S1. Socio-demographic and clinical characteristics of the sample, by diagnosis groups.

			Percentage or Mean ±Standard deviation							**Test**	**Post-hoc**
			**AN**				**Other**			*Non-corrected Corrected (sex and age)*	*Groups sharing the same letter do not differ (p≥0.050)*
			**AN-R**	**AN-BP**	**BN**	**BED**	**OSFED**	**UFED**			
Patients (N=131)		*Diagnosis:*	15.3%	9.9%	18.3%	29.0%	22.1%	5.3%	-	-	-
Sex		*Females:*	95.0%	100.0%	95.8%	86.8%	75.9%	100.0%		χ^2=9.88, p=0.078	
Age at assessment	*[years]*		24.3 ±9.08	21.7 ±5.92	29.1 ±11.40	41.0 ±13.86	37.7 ±16.60	33.1 ±10.61	*	F=8.06, p<0.001<br>F=7.75, p<0.001§	AN-R, AN-BP (a)<br>BN (ab)<br>UFED (abc)<br>OSFED (bc)<br>BED (c)
Schooling [6]		*Middle school:*	30.0%	61.5%	21.7%	14.7%	17.9%	14.3%		χ^2=20.00, p=0.171	
		High school:	45.0%	30.8%	52.2%	52.9%	53.6%	71.4%			
		Degree or more:	25.0%	7.7%	26.1%	32.4%	28.6%	14.3%			
School failures [62]		*Any:*	18.2%	25.0%	35.7%	40.0%	41.2%	33.3%		χ^2=2.06, p=0.873	
Relationship		*Single:*	65.0%	76.9%	75.0%	63.2%	62.1%	71.4%		χ^2=1.95, p=0.868	
		Couple:	35.0%	23.1%	25.0%	36.8%	37.9%	28.6%			
Housing [1]		*Alone:*	0.0%	0.0%	29.2%	29.0%	17.9%	14.3%	(*)	χ^2=34.92, p=0.029<br>LR-χ^2=19.74, p=0.474	
		New-family:	15.0%	7.7%	16.7%	39.5%	32.1%	28.6%			
		Old-family:	80.0%	92.3%	45.8%	26.3%	50.0%	57.1%			
		Other:	5.0%	0.0%	8.3%	5.2%	0.0%	0.0%			
Current occupation [1]		*Employed:*	25.0%	38.5%	58.3%	73.7%	67.9%	57.1%	(*)	χ^2=32.82, p=0.038<br>LR-χ^2=13.56, p=0.852	
		Student:	55.0%	61.5%	37.5%	13.2%	21.4%	28.6%			
		House-working:	5.0%	0.0%	0.0%	2.6%	0.0%	14.3%			
		Unemployed:	15.0%	0.0%	4.2%	5.3%	7.1%	0.0%			
		Retired:	0.0%	0.0%	0.0%	5.3%	3.6%	0.0%			
Age at FED onset [8]	*[years]*		16.8 ±2.61	18.5 ±7.21	14.6 ±4.78	20.1 ±13.29	18.9 ±9.28	17.6 ±9.94		F=1.11, p=0.358	
Duration of FED [8]	*[years]*		6.2 ±9.02	2.9 ±2.56	14.4 ±12.26	20.5 ±13.75	17.2 ±17.10	14.9 ±13.41	(*)	F=5.27, p<0.001<br>F=0.94, p=0.460	

		Percentage or Mean ±Standard deviation							**Test**	**Post-hoc**
		**AN**				**Other**			*Non-corrected*<br>*Corrected (sex and age)*	*Groups sharing the same letter do not differ ($p \geq 0.050$)*
		**AN-R**	**AN-BP**	**BN**	**BED**	**OSFED**	**UFED**			
BMI	*[kg/m²]*	17.4<br>±2.16	17.7<br>±1.08	22.9<br>±3.00	34.1<br>±7.68	26.6<br>±8.60	28.9<br>±13.59	*	F=22.77, p<0.001<br>F=14.32, p<0.001	AN-R (a)<br>AN-BP (ab)<br>BN (abc)<br>OSFED (bcd)<br>UFED (cd)<br>BED (d)
BMI (level)	*Obesity III:*	0.0%	0.0%	0.0%	26.3%	3.5%	14.3%	*	χ^2=145.71, p<0.001<br>LR-χ^2=129.96, p<0.001	-
	Obesity II:	0.0%	0.0%	0.0%	13.2%	27.6%	14.3%			
	Obesity I:	0.0%	0.0%	0.0%	29.0%	3.5%	14.3%			
	Overweight:	0.0%	0.0%	20.8%	21.1%	10.3%	0.0%			
	Normal range:	20.0%	23.1%	75.0%	10.5%	31.0%	42.9%			
	Mild thinness:	30.0%	38.5%	4.2%	0.0%	20.7%	0.0%			
	Moderate thinness:	25.0%	30.8%	0.0%	0.0%	3.5%	0.0%			
	Severe thinness:	25.0%	7.7%	0.0%	0.0%	0.0%	14.3%			
Previous hospitalizations (1)	*Any (for FED):*	25.0%	15.4%	0.0%	2.6%	17.9%	0.0%	*	χ^2=13.08, p=0.022<br>LR-χ^2=16.20, p=0.006	-
Psychiatric comorbidity	*Any (non-FED):*	20.0%	23.1%	29.2%	50.0%	41.4%	42.9%		χ^2=7.31, p=0.199	
Previous support (1)	*Any (mental health):*	75.0%	84.6%	70.8%	70.3%	62.1%	71.4%		χ^2=2.44, p=0.797	
Non-psychiatric comorbidity	*Any:*	25.0%	38.5%	41.7%	73.7%	48.3%	57.1%	(*)	χ^2=15.06, p=0.009<br>LR-χ^2=5.09, p=0.405	
	Chronic:	26.3%	33.3%	42.9%	73.0%	48.2%	57.1%	(*)	χ^2=14.01, p=0.013<br>LR-χ^2=3.13, p=0.679	
	Severe:	5.9%	8.3%	5.9%	20.6%	9.5%	0.0%	(*)	χ^2=21.67, p=0.016<br>LR-χ^2=12.00, p=0.285	
Antidepressant (2,4)	*Previous:*	15.0%	15.4%	25.0%	31.6%	18.5%	28.6%		χ^2=3.18, p=0.682	
	Current:	30.0%	38.5%	25.0%	54.1%	26.9%	14.3%		χ^2=9.25, p=0.098	
Stabiliser (2,4)	*Previous:*	5.0%	0.0%	16.7%	5.3%	3.7%	0.0%		χ^2=6.24, p=0.277	
	Current:	5.0%	7.7%	12.5%	10.8%	0.0%	28.6%		χ^2=6.99, p=0.205	
Antipsychotics (2,4)	*Previous:*	10.0%	0.0%	4.2%	10.5%	11.1%	0.0%		χ^2=3.09, p=0.700	
	Current:	20.0%	15.4%	8.3%	13.5%	15.4%	0.0%		χ^2=2.50, p=0.797	

		AN				Other			Test	Post-hoc
		Percentage or Mean ±Standard deviation							*Non-corrected*<br>*Corrected (sex and age)*	*Groups sharing the same letter do not differ (p≥0.050)*
		**AN-R**	**AN-BP**	**BN**	**BED**	**OSFED**	**UFED**			
Benzodiazepines [2,4]	*Previous:*	15.0%	7.7%	12.5%	23.7%	7.4%	14.3%		χ^2=4.19, p=0.533	
	Current:	25.0%	30.8%	8.3%	21.6%	15.4%	14.3%		χ^2=3.91, p=0.581	
Other drugs [2,4]	*Previous:*	5.0%	0.0%	20.8%	31.6%	14.8%	14.3%		χ^2=10.35, p=0.064	
	Current:	20.0%	15.4%	29.2%	29.7%	23.1%	28.6%		χ^2=1.66, p=0.901	
**EDI-3 specific scales**										
Eating Disorder Risk Composite	*[standard %ile]*	74.15<br>±14.579	92.85<br>±5.669	+93.21<br>±6.043	+88.34<br>±8.972	84.62<br>±12.040	74.14<br>±17.63	*	F=10.43, p<0.001<br>F=10.62, p<0.001	AN-R (a)<br>UFED (ab)<br>OSFED (bc)<br>AN-BP, BN, BED (c)
Drive for Thinness	*[standard %ile]*	81.00<br>±18.728	95.00<br>±5.958	+89.33<br>±9.823	73.53<br>±13.766	78.97<br>±15.042	68.29<br>±24.656	*	F=7.19, p<0.001<br>F=5.33, p<0.001	BED, UFED (a)<br>AN-R, OSFED (ab)<br>AN-BP, BN (b)
Bulimia	*[standard %ile]*	39.90<br>±24.402	86.38<br>±9.134	+94.88<br>±3.791	+92.50<br>±5.213	83.28<br>±19.943	70.14<br>±31.908	*	F=36.24, p<0.001<br>F=32.28, p<0.001	AN-R (a)<br>UFED (b)<br>AN-BP, OSFED (bc)<br>BN, BED (c)
Body Dissatisfaction	*[standard %ile]*	75.55<br>±16.891	89.08<br>±5.155	79.79<br>±13.606	82.24<br>±11.911	78.38<br>±14.644	71.86<br>±19.265	*	F=2.34, p=0.045<br>F=2.34, p=0.045	All groups (a)
**EDI-3 psychological scales**										
Global Psychological Maladjustment	*[standard %ile]*	65.85<br>±21.273	89.46<br>±7.378	69.17<br>±16.282	60.47<br>±21.109	65.90<br>±22.854	61.43<br>±29.944	*	F=4.13, p=0.002<br>F=2.71, p=0.024	AN-R (a)<br>BN, BED, OSFED, UFED (ab)<br>AN-BP (b)
Ineffectiveness Composite	*[standard %ile]*	77.85<br>±21.347	95.54<br>±3.455	78.12<br>±16.340	76.34<br>±20.801	76.48<br>±23.018	73.71<br>±26.887		F=2.11, p=0.068	
Interpersonal Problems Composite	*[standard %ile]*	72.20<br>±23.196	86.69<br>±11.842	71.29<br>±23.786	71.18<br>±24.592	73.00<br>±25.120	63.29<br>±22.860		F=1.21, p=0.307	
Affective Problems Composite	*[standard %ile]*	72.10<br>±23.179	94.85<br>±5.161	79.83<br>±17.490	67.63<br>±22.061	72.90<br>±22.109	70.71<br>±26.380	(*)	F=3.81, p=0.003<br>F=2.26, p=0.052	
Overcontrol Composite	*[standard %ile]*	72.85<br>±21.465	89.15<br>±9.299	80.08<br>±13.916	67.21<br>±21.115	77.41<br>±22.641	65.86<br>±38.299	*	F=2.96, p=0.015<br>F=2.39, p=0.042	All groups (a)

		Percentage or Mean ±Standard deviation							**Test**	**Post-hoc**
		**AN**				**Other**			*Non-corrected*<br>*Corrected (sex and age)*	*Groups sharing the same letter do not differ (p≥0.050)*
		**AN-R**	**AN-BP**	**BN**	**BED**	**OSFED**	**UFED**			
Low Self-Esteem	*[standard %ile]*	77.65<br>±18.135	94.54<br>±4.612	73.83<br>±21.050	73.97<br>±22.291	74.69<br>±22.694	71.29<br>±28.418		F=2.28, p=0.051	
Personal Alienation	*[standard %ile]*	74.25<br>±26.715	94.69<br>±3.728	78.58<br>±21.092	74.45<br>±23.524	75.28<br>±24.956	73.00<br>±29.126		F=1.76, p=0.126	
Interpersonal Insecurity	*[standard %ile]*	69.85<br>±25.936	86.38<br>±9.403	69.83<br>±23.392	67.00<br>±27.219	71.38<br>±26.408	51.43<br>±26.038		F=2.03, p=0.078	
Interpersonal Alienation	*[standard %ile]*	69.70<br>±22.309	80.38<br>±20.222	64.62<br>±30.020	69.53<br>±23.241	68.10<br>±26.994	71.71<br>±18.409		F=0.71, p=0.617	
Interoceptive Deficits	*[standard %ile]*	73.70<br>±23.756	96.85<br>±2.577	81.58<br>±19.978	71.87<br>±21.261	75.83<br>±21.824	77.86<br>±22.289	(*)	F=3.23, p=0.009<br>F=2.07, p=0.073	
Emotional Dysregulation	*[standard %ile]*	65.80<br>±21.618	85.85<br>±12.233	68.25<br>±19.753	53.32<br>±27.876	62.52<br>±25.131	51.71<br>±28.277	(*)	F=4.25, p=0.001<br>F=2.20, p=0.059	
Perfectionism	*[standard %ile]*	60.80<br>±27.358	74.62<br>±20.569	66.67<br>±23.360	56.84<br>±25.863	65.48<br>±29.907	63.14<br>±33.899		F=1.07, p=0.381	
Ascetism	*[standard %ile]*	74.35<br>±26.268	91.85<br>±7.470	81.83<br>±15.920	70.84<br>±20.289	78.83<br>±21.752	64.86<br>±36.603	(*)	F=2.73, p=0.022<br>F=2.12, p=0.067	
Maturity Fears	*[standard %ile]*	61.20<br>±31.557	82.38<br>±18.369	52.08<br>±31.931	53.29<br>±31.554	49.83<br>±34.566	48.57<br>±30.193	(*)	F=2.40, p=0.041<br>F=1.63, p=0.157	
**RAADS-R (with sub-scales)**										
Possible ASD (above cut-off)	*High score (≥65):*	35.0%	69.2%	62.5%	52.6%	58.6%	28.6%		χ^2=6.89, p=0.235	
Total score	*[score: 0, 240]*	61.81<br>±41.856	90.94<br>±41.759	75.48<br>±34.032	73.81<br>±38.576	80.39<br>±41.977	47.24<br>±23.145		F=1.72, p=0.135	
Social relatedness scale	*[score: 0, 117]*	31.84<br>±21.383	39.60<br>±19.534	37.24<br>±19.291	36.33<br>±21.042	40.91<br>±23.353	22.08<br>±11.729		F=1.19, p=0.316	
Circumscribed interests scale	*[score: 0, 42]*	14.20<br>±9.961	20.95<br>±8.091	17.44<br>±8.055	15.17<br>±8.744	17.62<br>±8.027	10.71<br>±6.601		F=1.98, p=0.086	
Language scale	*[score: 0, 21]*	3.40<br>±3.705	7.38<br>±5.173	4.14<br>±3.930	5.22<br>±4.130	4.90<br>±4.161	2.86<br>±3.024		F=2.01, p=0.081	
Sensory–Motor scale	*[score: 0, 60]*	12.28<br>±12.058	22.97<br>±14.805	16.63<br>±10.645	17.10<br>±11.961	16.95<br>±11.546	11.68<br>±7.727		F=1.56, p=0.177	

		Percentage or Mean ±Standard deviation						Test	Post-hoc
		**AN**				**Other**		*Non-corrected*	*Groups sharing the same letter*
		**AN-R**	**AN-BP**	**BN**	**BED**	**OSFED**	**UFED**	*Corrected (sex and age)*	*do not differ (p≥0.050)*
**CAT-Q (with sub-scales)**									
Camouflage (above cut-off)	*High score (≥100):*	25.0%	46.2%	20.8%	26.3%	17.2%	28.6%	χ²=4.31, p=0.516	
Total score	*[standard z]*	-0.12 ±1.459	+0.99 ±1.267	+0.11 ±1.156	+0.04 ±1.647	-0.10 ±1.450	+0.04 ±1.218	F=1.21, p=0.307	
Compensation score	*[standard z]*	-0.70 ±0.862	+0.41 ±1.256	-0.32 ±1.011	-0.19 ±1.361	-0.39 ±1.209	-0.28 ±1.011	F=1.51, p=0.192	
Masking score	*[standard z]*	-0.35 ±1.437	+0.78 ±1.092	+0.19 ±0.953	-0.11 ±1.601	-0.07 ±1.384	+0.25 ±1.225	F=1.31, p=0.263	
Assimilation score	*[standard z]*	+0.67 ±1.610	+1.10 ±1.089	+0.38 ±1.100	+0.34 ±1.586	+0.22 ±1.333	+0.11 ±0.927	F=0.96, p=0.443	
**Other ASD-related**									
Adult Autism Spectrum Quotient (13)	*[score: 0, 50]*	18.9 ±8.92	24.4 ±9.39	20.4 ±8.08	20.3 ±7.01	21.6 ±6.40	13.0 ±4.94	F=2.11, p=0.069	
Empathy Quotient (3)	*[score: 0, 80]*	46.3 ±11.53	49.4 ±13.88	44.9 ±10.12	43.7 ±12.68	41.5 ±10.78	51.9 ±12.27	F=1.48, p=0.202	

Footnotes: %ile: Percentile; **AN:** Anorexia Nervosa; **ASD:** Autistic Spectrum Disorder; **BED:** Binge-Eating Disorder; **BMI:** Body Mass Index; **BN:** Bulimia Nervosa; **-BP:** Binge-Eating/Purging sub-type of AN; **CAT-Q:** Camouflaging Autistic Traits Questionnaire; **EDI-3:** Eating Disorder Inventory, version 3; **FED:** Feeding or Eating Disorder; **LR:** Likelihood Ratio; **OSFED:** Other Specified FED; **Other:** Diagnosis group including OSFED and UFED; **-R:** Restrictive sub-type of AN; **RAADS-R:** Ritvo Autism Asperger Diagnostic Scale, Revised version; **UFED:** Unspecified FED. ***:** Comparison between diagnosis groups showed a statistically significant difference (p<0.050); **(*):** Comparison between diagnosis group was no more statistically significant (p≥0.050) after the correction for sex and age. **(N):** The numbers of missing observations are indicated in superscript, in parentheses. **§:** Only the correction by sex was adopted.

Figure S1. Parallel mediation effects in the overall sample (N=131) and within each diagnostic group. Direct prediction was of RAADS-R score on GPMC scale (EDI-3, in standard percentiles). Indirect effect includes both mediation effects of CAT-Q score (in standard z-score) and EDRC scale (EDI-3, in standard percentiles). On the **left**, analyses were not adjusted for any confounder. On the **right**, all analyses were adjusted for potential confounders: age at assessment (in years) and BMI (in kg/m²).

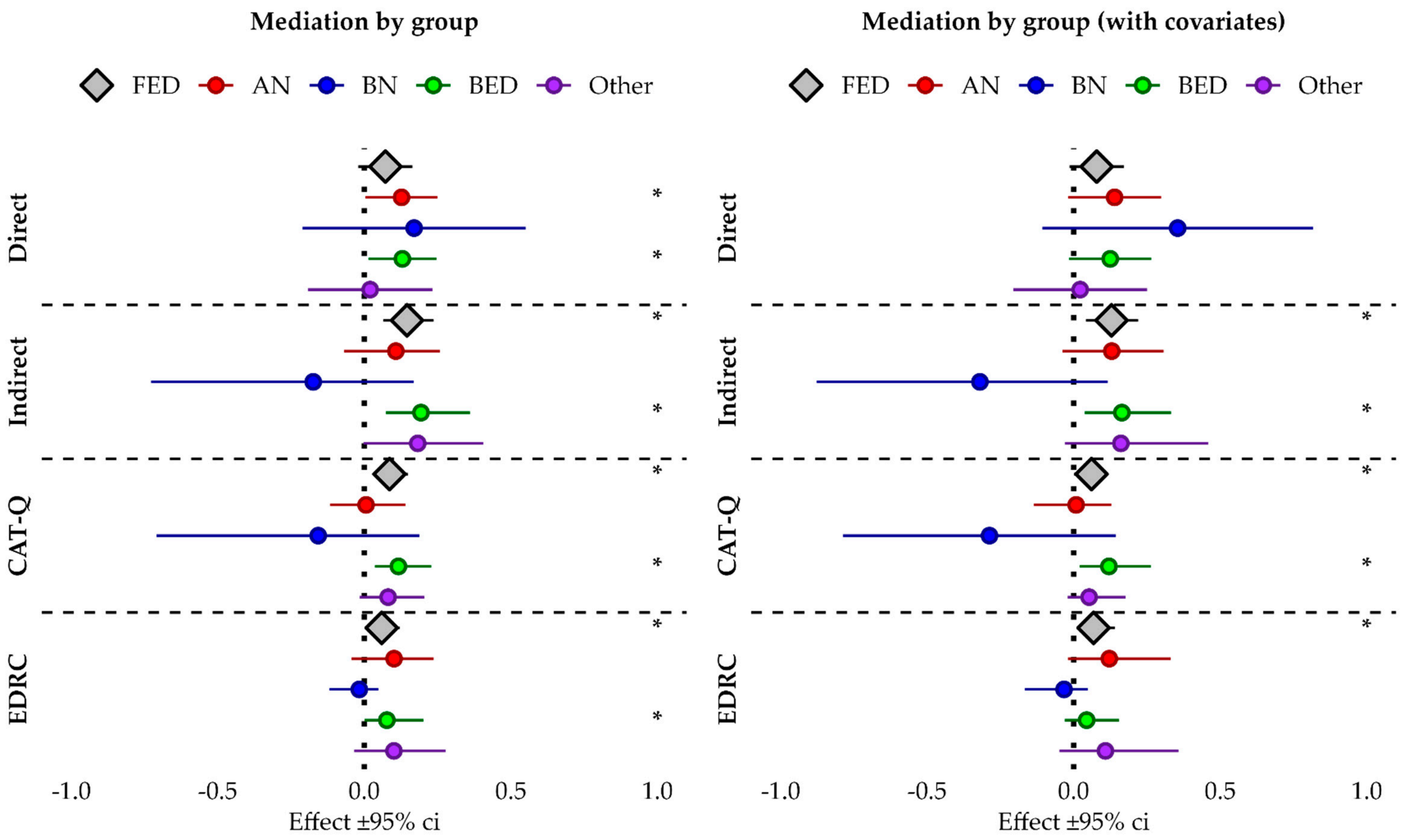

Footnotes: AN: Anorexia Nervosa; **BED:** Binge-Eating Disorder; **BMI:** Body Mass Index; **BN:** Bulimia Nervosa; **CAT-Q:** Camouflaging Autistic Traits Questionnaire; **ci:** Confidence Interval; **EDI-3:** Eating Disorder Inventory, version 3; **EDRC:** Eating Disorder Risk Composite score (EDI-3); **FED:** Feeding or Eating Disorder (including all diagnosis groups); **GPMC:** Global Psychological Maladjustment Composite scale (EDI-3); **OSFED:** Other Specified FED; **Other:** Diagnosis group including OSFED and UFED; **RAADS-R:** Ritvo Autism Asperger Diagnostic Scale, Revised version; **UFED:** Unspecified FED. ***:** Statistically significant ($p<0.050$).
